# Thresholds in marsh resilience to the *Deepwater Horizon* oil spill

**DOI:** 10.1038/srep32520

**Published:** 2016-09-28

**Authors:** Brian R. Silliman, Philip M. Dixon, Cameron Wobus, Qiang He, Pedro Daleo, Brent B. Hughes, Matthew Rissing, Jonathan M. Willis, Mark W. Hester

**Affiliations:** 1 Division of Marine Science and Conservation, Nicholas School of the Environment, Duke University, 135 Duke Marine Lab Road, Beaufort, NC 28516, USA; 2 Department of Statistics, Snedecor Hall, Iowa State University, Ames, IA 50011-1210, USA; 3 Abt Associates, 1881 Ninth Street, Suite 201, Boulder, CO 80302, USA; 4 Instituto de Investigaciones Marinas y Costeras (IIMyC), UNMDP, CONICET, CC1260 Correo Central, B7600WAG, Mar del Plata, Argentina; 5 Department of Ecology and Evolutionary Biology, University of California, Santa Cruz, 100 Shaffer Road, Santa Cruz, CA 96060, USA; 6 Institute for Coastal and Water Research, Department of Biology, University of Louisiana at Lafayette, Lafayette, LA 70504, USA

## Abstract

Ecosystem boundary retreat due to human-induced pressure is a generally observed phenomenon. However, studies that document thresholds beyond which internal resistance mechanisms are overwhelmed are uncommon. Following the *Deepwater Horizon* (DWH) oil spill, field studies from a few sites suggested that oiling of salt marshes could lead to a biogeomorphic feedback where plant death resulted in increased marsh erosion. We tested for spatial generality of and thresholds in this effect across 103 salt marsh sites spanning ~430 kilometers of shoreline in coastal Louisiana, Alabama, and Mississippi, using data collected as part of the natural resource damage assessment (NRDA). Our analyses revealed a threshold for oil impacts on marsh edge erosion, with higher erosion rates occurring for ~1–2 years after the spill at sites with the highest amounts of plant stem oiling (90–100%). These results provide compelling evidence showing large-scale ecosystem loss following the *Deepwater Horizon* oil spill. More broadly, these findings provide rare empirical evidence identifying a geomorphologic threshold in the resistance of an ecosystem to increasing intensity of human-induced disturbance.

Human-driven environmental changes such as global warming, pollution, overharvesting, and acidification have increased the amount and number of stressors ecosystems experience, leading to large declines in many marine, freshwater, and terrestrial communities^1^,^2^. The response of ecosystems to these stressors varies from strong resistance to drastic declines in habitat coverage to transformation into an alternate stable state^3^,^4^,^5^. In the southeastern United States, for example, the combination of extreme drought and intensified consumer grazing pressure has generated threshold levels of stress and subsequent die-off of large areas of marsh plant communities^6^. The system has recovered in most areas, however, and has not transitioned into an alternate stable state, most likely because powerful positive interactions and spatial processes facilitate plant recolonization of denuded tidal flats^7^.

One key to conserving and protecting these threatened ecosystems is to identify and guard against the exceedance of ecosystem thresholds – the point at which a small change in an environmental stressor produces a cascading decline in ecosystem cover and health^8^. Although a significant body of theoretical and modeling evidence suggests that thresholds in ecosystem resistance to disturbance are likely to occur in many habitats^9^, empirical data that link these inflection points to specific levels of large-scale disturbances are rare (but see ref. 10).

For many estuarine ecosystems, especially those governed by biogeomorphic feedbacks, habitat edges are the nexus for ecosystem growth and decline. Both biotic and abiotic stressors, such as consumer fronts^11^ and wave stress^12^, are typically concentrated at habitat edges, and these edges are often the primary point of habitat decline if the edge is in retreat^13^,^14^. In the Gulf of Mexico, for example, extremely rapid marsh loss (40 km^2^ yr^−1^ from 1985 to 2010)^15^ is driven by the synergistic impacts of decreased sedimentation from land-use change and river channelization, subsidence-enhanced relative sea-level rise, and changes in hydrologic connectivity^16^,^17^. Marsh edge erosion rates are particularly high in these areas^18^, raising concern as to how an already stressed system may respond to a major disturbance.

In the summer of 2010, oil released from the DWH disaster was found on more than 700 km of salt marsh shorelines across the Gulf of Mexico^19^. Given the microtidal regime of the Gulf, this oil was generally most concentrated along the marsh edge, often visible as a black belt along the shoreline that was ~5–15 meters in width^3^. Oiling of marshes from the DWH spill thus created a concentrated disturbance on the ecosystem’s already stressed edge. Field studies at a limited number of sites indicated that heavy levels of oiling led to elevated erosion rates, most likely because plant death resulted in decreased soil strength and reduced resistance to wave erosion^3^,^4^,^20^,^21^. A substantial amount of the marsh shoreline oiling, however, was characterized as light to intermediate^19^, and little is known about the impact of oiling on marsh edge erosion across a range of oiling levels.

In this study, we examined the relationship between plant stem oiling and marsh erosion rates Gulf-wide to test for thresholds in the functional relationship between degree of plant oiling and erosion rates. We analyzed data collected for the Coastal Wetland Vegetation (CWV) survey as part of the NRDA process^22^. Data were collected across 103 sites (Fig. 1) across the states of Louisiana, Alabama, and Mississippi and spanning 5 categories of oiling defined by the percentage of stem height oiled (0%, 0.1–10%, 10.1–50%, 50.1–90% or 90.1–100%; here after, 0%, 0–10%, 10–50%, 50–90%, 90–100%). At each site, cumulative erosion was monitored based on field surveys of marsh retreat relative to a datum established in fall 2010 (Louisiana sites) or spring 2011 (Alabama and Mississippi sites) and remeasured in fall 2011, fall 2012 and fall 2013. As previous field experiments have shown that: (1) accelerated erosion rates are associated with belowground plant mortality^3^, and (2) mortality of both aboveground^23^ and belowground biomass^3^,^24^ are pronounced at the heaviest oiling levels, our hypotheses were that erosion rates would be positively correlated with degree of plant oiling, and that this response might exhibit threshold behavior at the highest (90–100%) stem oiling level.

## Results

**Figure 2:**
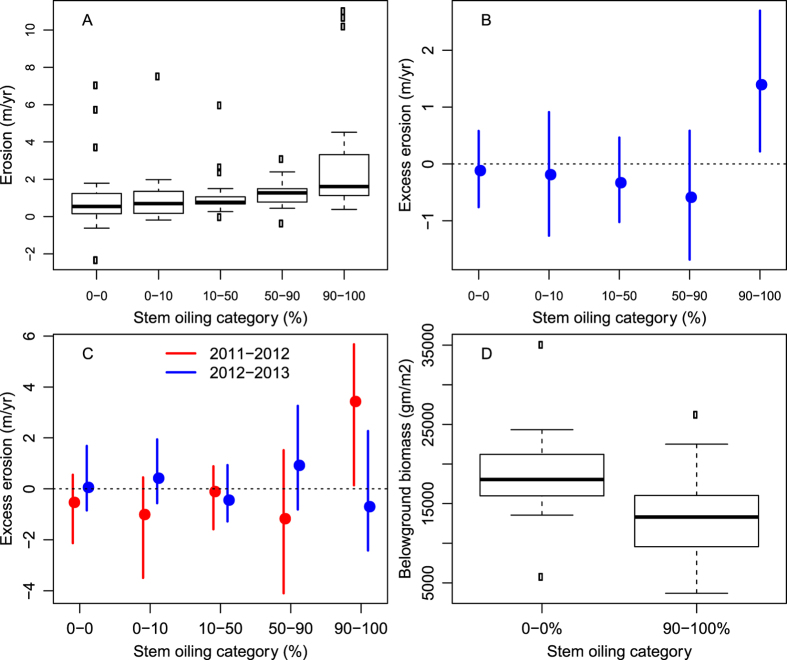
(**A**) Box plots of *unadjusted* erosion rates (m/yr) in each stem oiling category for all sites. (**B**) Mean excess erosion (m/yr) for each stem oiling category. Excess erosion is the difference between the observed mean erosion for that stem oiling category and the expected mean *wave-adjusted* erosion rate if there were no differences in erosion among the stem oiling categories. The vertical lines are the central 95% randomization distributions for excess erosion in each stem oiling category. When the vertical line does not cross 0, the *p*-value for the comparison of that stem oiling category to the overall erosion rate is less than 0.05. (**C**) Mean excess erosion (m/yr) by stem oiling category in 2011–2012 and 2012–2013 for all sites. Symbols as in Fig. 2B. (**D**) Box plot comparing total belowground biomass between the 0% and 90.1–100% oiling categories in CWV plots nearest to the marsh edge in Fall 2010.

Analyzing all sites across the three states in spring 2011 and fall 2013 (total sample sizes ranged from 96 to 103 sites; see Methods), we detected differences in erosion rate among the stem oiling levels (Kruskal-Wallis; p = 0.0021). Specifically, the total erosion in marshes with 90–100% stem oiling was more than three times higher than in marshes with no stem oiling (Fig. 2A; Table S1A). When we accounted for potential confounding effects of variation in wave exposure among marsh sites, the mean erosion rate in the 90–100% stem oiling level was 1.4 m/yr higher than expected, while the mean erosion rates in the other four stem oiling levels were lower than expected (Fig. 2B; Table S1B). Examination of annualized erosion rates revealed that erosion at sites with the highest stem oiling levels was 3.4 m/yr larger than expected (p = 0.027) during the 2011–2012 survey period, but was indistinguishable from other oiling levels during 2012–2013 (Fig. 2C; Table S2).

We observed the same patterns when the analysis was restricted only to sites in Louisiana, which had a longer sampling period and comprised the majority of our study sites (see Methods). The mean cumulative (2010–2013) erosion rate in the 90–100% stem oiling level was 4.0 m/yr, significantly higher than mean erosion rates of 1.4 to 2.1 m/yr in the other oiling levels (Kruskal-Wallis; p = 0.033; Figure S1A). After adjustment for wave energy, the erosion rate in the 90–100% stem oiling level was 1.6 m/yr more than expected, but this difference was not statistically significant (p = 0.13). Using just the Louisiana sites, on a year-by-year basis, mean wave-adjusted erosion rates in the highest stem oiling level were 1.6 m/yr higher than expected from fall 2010–fall 2011 (p = 0.040; Figure S2, Table S4). For fall 2011–fall 2012, the difference was 3.0 m/yr (p = 0.068). By the third year of the study (fall 2012–fall 2013), erosion rates for the highest oiling level were no longer elevated relative to the other levels.

To evaluate whether accelerated erosion at high stem oiling sites was correlated with loss of belowground biomass at those sites, we compared total belowground biomass in unoiled vs heavily oiled sites for the fall 2010 sampling event. This sampling date in 2010 was ~5 months after the oiling occurred, but before heavily oiled marsh edges were lost to erosion and the marsh platform lowered to elevations where marsh plants drown^3^,^23^. In fall 2010, the mean total belowground biomass in the sites with the heaviest (90–100%) stem oiling was approximately 13,000 g/m^3^, ~30% lower than the mean total belowground biomass in the unoiled sites (~19,000 g/m^3^, p = 0.036; Fig. 2D). Because the NRDA protocol for measuring total belowground biomass included both live and dead root material, this analysis should be considered a conservative estimate of how oiling influenced the live plant roots that are critical for providing soil strength in these environments. Another recent study^24^ that evaluated live belowground biomass as a function of qualitative oiling severity found significant reductions in live belowground biomass at the heaviest oiling levels.

**Figure 3:**
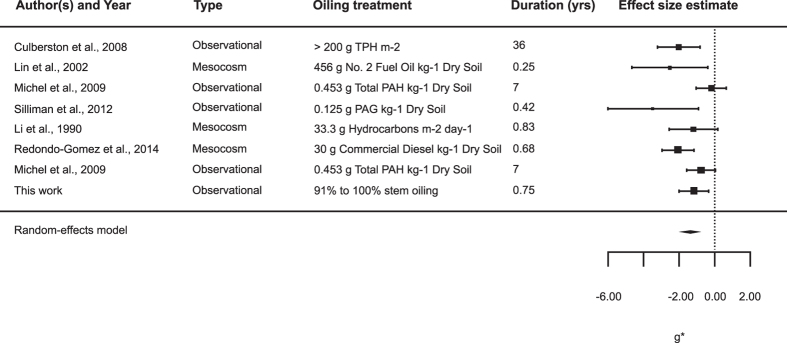
Meta-analysis results testing for the mean effect of oiling on *Spartina* spp. belowground biomass. The mean effect size (Hedges’ *g* ± 95% *CI*) are reported, with negative values indicating negative effects of oiling on belowground biomass. 95% CI that do not overlap with the zero line indicate significant effects.

Consistent with field evidence for reduced belowground biomass following the DWH spill, a meta-analysis of seven published studies investigating oiling impacts on *Spartina* spp. also revealed a consistent pattern of negative impacts of heavy oiling on belowground plant biomass. Averaged across all studies, we found a significantly negative effect of oiling on *Spartina* spp. belowground biomass (Hedges’ *g* = −1.379, 95% *CI* = −1.99 to −0.77, *P* < 0.0001; Fig. 3).

## Discussion

The results of our regional-scale analysis reveal that accelerated marsh erosion reported in small-scale studies^3^,^20^,^25^ after the DWH spill also occurred extensively across the larger area of impacted coastal marshes. We observed this effect even when erosion rates were adjusted to correct for the effects of wave exposure on marsh edge erosion. Our analysis across the full range of stem oiling levels further demonstrated that Gulf Coast salt marsh ecosystems exhibited a threshold behavior in response to increasing oil disturbance: there was no observable difference in erosion rate relative to unoiled marshes at the low to moderately high stem oiling levels, but statistically significant increases in erosion rate at the highest stem oil levels (90–100% stem oiling). Because past experimental transplant studies at similarly eroded areas along marsh edges have shown that marsh plants cannot regrow due to inundation stress (i.e., after erosion the marsh platform goes from being intertidal to being primarily submerged)^3^, it is very likely that these disturbance-enhanced erosion rates have led to irreversible land loss along affected marsh edges of the Gulf of Mexico. However, to fully assess whether a tipping point, and not just a threshold was reached, we suggest that plant recovery patterns at these sites should be further monitored.

It is very likely that the death of belowground plant material at the highest levels of stem oiling triggered the observed increases in erosion rate at the heavily oiled sites. This contention is supported by: 1) past studies that demonstrate reductions in soil strength and/or irreversible marsh platform erosion when the belowground root network is compromised on eroding marsh edges^3^,^24^; 2) previous studies that have documented significant die-back of belowground plant material as a result of heavy oiling, as revealed in our meta-analysis; and 3) vegetation survey results from the CWV survey that evince a significantly negative effect of heavy oiling on both aboveground^23^ and belowground biomass (Fig. 2D). The high level of resistance these marshes displayed to oil-induced, elevated erosion is predicted by past studies that have assessed potential for oiling impacts with small-scale oil addition studies in wetlands^25^,^26^. In these studies, belowground plant material – which is anticipated to be the key component underlying the protective effects of marshes – was resilient to oiling and only exhibited complete mortality in heaviest oiling treatments – a pattern we also observed in our study.

The combination of our regional-scale survey results with many small-scale, comparative studies indicating that the presence of live belowground plant material reduces erosion stress along marsh edges, challenges a recent experimental study^27^, which concluded that wetland plants do not protect against shoreline erosion. Resolving whether the wetland-protective paradigm is indeed robust, as suggested by the results of our large-scale survey, or should be reconsidered, as suggested by this experimental study^27^, will require further experimental studies conducted at larger scales that test for context dependency of plant protective effects and the relative contribution of belowground and aboveground plant material to any effect detected.

Approximately 18 months after the DWH spill, the increased erosion rates in the highest stem oiling level were no longer detected. This observed cessation of elevated erosion rates is consistent with results from a more process-oriented, but smaller scale study of the impacts of heavy oiling on shoreline erosion rates following the DWH disaster^3^. It is also consistent with plant health observations^23^,^26^,^28^,^29^, where the majority of significant reductions to plant health and productivity due to oiling occurred through 2012. Return to background erosion rates thus likely occurred as the erosive front intersected live root mats generated from re-growing and/or surviving areas of marsh plants that were not as severely impacted by oiling. This cessation of elevated erosion rates does not erase the accelerated and permanent loss of marshes that occurred for ~ 1.5 years.

## Conclusions

Our results provide regional-scale evidence of a threshold in oil impact beyond which the resilience of salt marsh ecosystems was compromised, leading to large-scale land loss in the Gulf of Mexico, with a strong potential for irreversible ecosystem collapse, i.e. a tipping point. The resistance threshold of marshes to oil-induced, elevated erosion rates was high, non-linear and occurred at 90–100% stem oiling. This high level of resistance was likely driven by the demonstrated tolerance of belowground plant material to light and moderate levels of oiling. Empirical data such as these that identify levels of stress at which an ecosystem’s threshold to disturbance occurs are uncommon, but are critical to understand to better protect and restore valuable coastal systems in the face of global environmental change^30^. Given the elevated importance of salt marshes as part of a first line of defense against the rising seas^6^,^31^,^32^, our study highlights the need for a more robust understanding of salt marsh edge dynamics and their interactions with intensifying human-induced stressors.

## Materials and Methods

### Field methods

The CWV survey included multiple types of marsh. Here, we analyzed erosion data only from mainland herbaceous marshes, as 1) our previous experimental and comparative studies were also conducted in mainland herbaceous marshes^3^,^23^, 2) this marsh type was commonly impacted by oiling, and 3) using one marsh type controlled as much as possible for many biotic and abiotic conditions that can vary across different types of wetlands in this region (e.g. salinity, plant species, and tidal range). Mainland herbaceous marshes sampled during the CWV study were located primarily along the inland edges of protected bays and estuaries, and were dominated by *Spartina alterniflora* in Louisiana^23^ and co-dominated by *Juncus roemerianus* and *S. alterniflora* in Mississippi and Alabama^28^. The initial CWV survey of Louisiana marsh sites was conducted based on a stratified random sample of 78 sites from a collection of 713 marsh pre-assessment survey sites^23^. One of the 78 CWV sites was missing data in fall 2012 and fall 2013; the final Louisiana dataset described here therefore included 77 of the 78 CWV sites. Seven of the Louisiana sites were missing erosion data in spring 2011.

The initial CWV dataset, established during fall 2010, was supplemented in spring 2011 with 26 sites in Mississippi and Alabama, which were selected from an additional 114 pre-assessment sites in those states. Four of these sites were missing data in fall 2011. The complete three-state CWV database contained a total of 103 sites, although some analyses were based on a slightly smaller sample sizes because of the missing values. In all cases, strata were defined by the extent of stem oiling observed during the pre-assessment survey (0%, 0.1–10%, 10.1–50%, 50.1–90%, and 90.1–100%), which occurred between late May and early September of 2010^23^,^28^.

Measurements of marsh edge position were made at all sites in fall 2011, fall 2012, and fall 2013. We focused our analysis on annual and cumulative erosion between spring 2011 and fall 2013 at all sites in the CWV survey, because this was the longest period with data from all three states. We also analyzed annual erosion from fall 2010 to fall 2013 for the Louisiana sites, which provides additional insight into the effects of oiling on erosion in the first six months following the spill.

At each site, survey teams established marsh edge and inland stakes using polyvinyl chloride (PVC) poles to demarcate the beginning and end of a line transect perpendicular to the shoreline. The shoreline stake was placed at the marsh edge and the inland stake was placed at the furthest inland point of oiling documented either during the pre-assessment survey or transect installation. At unoiled reference sites, the inland stake was placed at 20 m inland from the marsh edge. On each sampling date, survey teams measured the distance from the inland stake to the current marsh edge to determine cumulative erosion. Complete details on the CWV survey design and field observations can be found in (ref. 22).

In order to account for effects of incident wave energy on erosion rates, we used a measure of relative wind-wave exposure that was quantified for each of the sites^33^,^34^. This metric, referred to as the mean wave exposure index, is calculated from the product of average wind speed and fetch along each of 8 cardinal directions. A detailed description of how the wave exposure index was calculated can be found in ref. (34).

### Erosion data analysis

Differences in marsh erosion rate among stem oiling levels were evaluated using Kruskal-Wallis tests^34^ and the analysis of means^36^,^37^. The Kruskal-Wallis test evaluates any difference between stem oiling levels, whereas the analysis of means detects whether one group differs from the others. Both methods are non-parametric procedures because the data did not meet the normality or equal variance assumptions of analysis of variance (ANOVA). For the Kruskal-Wallis statistic, *R*_*i*_ and *n*_*i*_ were the average rank and sample size for each stem oiling level, respectively. The analysis of means^36^ compares the erosion rate for each stem oiling level to the overall erosion rate in all sites. The nonparametric version of analysis of means^37^ is based on ranks for each stem oiling level. The null distribution of the test statistic was evaluated by randomization, using 9999 randomizations of stem oiling values to erosion values. Differences were considered significant at the level *P* < 0.05. No adjustment for multiple testing was made because of the *a-priori* expectation that the differences would be largest in the 90–100% stem oiling level.

To address the influence of variation in wave exposure among marsh sites, we repeated these analyses after grouping observations by similar wave energy. This approach is an extension of the Skillings-Mack method^38^. Breakpoints between wave energy groups were set at the deciles of mean wave energy, giving 10 groups. Measured erosion rates were then ranked within each wave energy group. We computed the mean and median erosion in each stem oiling level and compared those values to distributions obtained by randomly reassigning erosion values to stem oiling levels within each wave energy group. Mean excess erosion for each stem oiling level was computed as the difference between the mean observed erosion and the mean of the randomly reassigned erosion values. *P*-values and 95% confidence intervals for excess erosion were obtained by randomly permuting erosion values within each wave-energy block using 9999 randomizations. We repeated the analysis using 5 and 15 groups of wave energy. Our results were insensitive to the number of wave energy groups, so we present here only the results from analyses using 10 wave energy groups.

### Effects of oiling on belowground biomass

The CWV sampling design did not partition total belowground biomass into live and dead components. Thus we do not have direct observational evidence of the impact of oiling on live belowground biomass. In an effort to address this data gap, we followed two approaches. First, we summarized the effects of heavy oiling on total belowground biomass as measured in the CWV study. The belowground biomass in control and heavily oiled Louisiana sites was compared using a t-test. Because one CWV site outside of Louisiana was heavily oiled and there was a regional trend in belowground biomass, we only analyzed belowground biomass in Louisiana. Since total belowground biomass included both live and dead components, this analysis provides a conservative estimate of how oiling might have influenced the live plant roots that are important to soil strength in these habitats. In addition, we conducted a meta-analysis of published observational and experimental studies investigating oiling v. control differences in belowground biomass of the marsh plant genus *Spartina*. We used a Web of Science literature search from September 16, 2015 to assemble the existing studies investigating oiling impacts on *Spartina* belowground biomass using the following search criteria: TS = Spartina AND oil* or oil spill* AND belowground. Our Web of Science search generated 122 publications, and out of those we narrowed the total number of studies to six^3^,^39^,^40^,^41^,^42^ that included oil impacts to *Spartina* belowground biomass by either measuring plant live belowground biomass or plant total (i.e., live + dead) belowground biomass. Although our objective was to evaluate the effect of oiling on live belowground biomass, we believe our inclusion of studies that measured total belowground biomass also makes our meta-analysis conservative, since these studies may underestimate the effect of oiling on live biomass. One study^41^ investigated two species of *Spartina*, generating a total sample size of *n* = 7.

For each study, we extracted the mean *Spartina* belowground biomass for oiled and unoiled treatments, along with the standard deviation and sample size. We then used the extracted data to measure the standardized effect size by calculating Hedges’ *g*^43^ using the *metafor* package^44^ in R^45^. A negative Hedges’ *g* value where the 95% CI did not overlap zero indicates as significantly negative effect from oiling. We determined the overall mean effect across all studies using a random-effects model. When studies used multiple treatments (e.g., unoiled, light, medium, and high oiling) we used only the endpoint treatments (i.e., no oiling and high oiling) to look at the maximum possible effects. When multiple core depths were used as a factor in a single study we only used belowground responses of the shallowest layers because we assumed that oiling would have the greatest impact at shallower sediment depths.

## Additional Information

**How to cite this article**: Silliman, B. R. *et al*. Thresholds in marsh resilience to the *Deepwater Horizon* oil spill. *Sci. Rep*. **6**, 32520; doi: 10.1038/srep32520 (2016).

## Supplementary Material

Supplementary Information

## Figures and Tables

**Figure 1:**
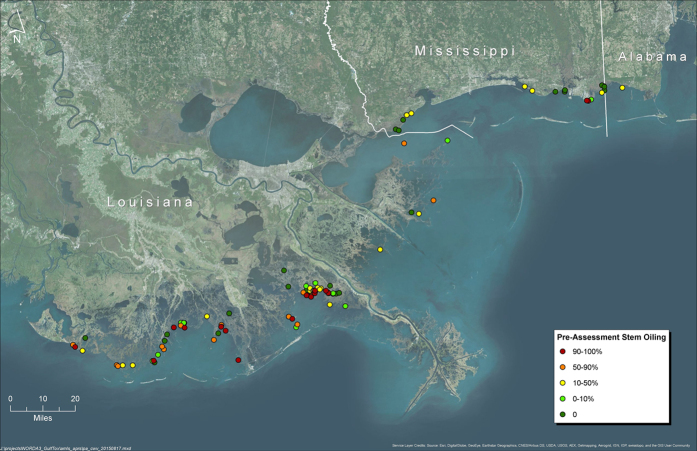
Map of all survey sites. Figure created using ArcGIS, 10.3, (http://desktop.arcgis.com/en/arcmap/).
