# Supplementary material for: Thresholds in marsh resilience to the Deepwater Horizon oil spill
Source: Sci Rep. 2016 Sep 28;6:32520. doi: 10.1038/srep32520 (PMC5040145; doi:10.1038/srep32520)
Supplement: Supplementary Information [file srep32520-s1.doc]

**Supplementary Information**

**Table S1A** Summary of the data for Figure 2A. *P*-values are for the nonparametric Analysis of Means method, which compares the median erosion rate for each stem oiling group to the overall median erosion rate.

| Stem oiling | *n* | Erosion rates (m/yr) | | | *P*-value |  |
| --- | --- | --- | --- | --- | --- | --- |
| category |  | Mean | SE | Median |  |  |
| 0% | 28 | 1.0 | 0.3 | 0.5 | 0.0076 | |
| 0.1–10% | 14 | 1.2 | 0.5 | 0.7 | 0.14 | |
| 10.1–50% | 24 | 1.1 | 0.2 | 0.8 | 0.26 | |
| 50.1–90% | 14 | 1.3 | 0.2 | 1.3 | 0.095 | |
| 90.1–100% | 16 | 3.3 | 0.9 | 1.6 | 0.0001 | |
| All sites | 96 | 1.5 |  |  |  |  |

**Table S1B** Summary data for Figure 2B, after adjusting erosion rates for wave exposure. The “expected mean, no effect of oil” represents the expected mean erosion rates when stem oiling categories are randomly assigned to sites within each wave exposure group. The “change from random expectation” values are the difference between the observed mean erosion rate and the expected mean erosion rate. The *P*-value is for the nonparametric analysis of means, comparing the median erosion rate for each stem oiling group to the overall median erosion rate, after adjusting for wave exposure.

| Stem oiling |  | Erosion rates (m/yr) | | |  |
| --- | --- | --- | --- | --- | --- |
| category | n | Mean erosion rate | Expected mean, no effect of oil | Change from random expectation | *P*-value |
|  |  |  |  |  |  |
| 0% | 28 | 1.0 | 1.1 | -0.1 | 0.10 |
| 0.1–10% | 14 | 1.2 | 1.4 | -0.2 | 0.34 |
| 10.1–50% | 24 | 1.1 | 1.4 | -0.3 | 0.35 |
| 50.1–90% | 14 | 1.3 | 1.9 | -0.6 | 0.400 |
| 90.1–100% | 16 | 3.3 | 1.9 | 1.4 | 0.0048 |

**Table S2** Summary data for Figure 2C. Analog of table S 1B with year-specific results for all sites: mean erosion.

| 2011–2012 | | | | | | |
| --- | --- | --- | --- | --- | --- | --- |
| Stem oiling category | *n* | Mean erosion | Expected when no difference | Change from expectation | | *P*-value |
| 0% | 27 | 1.1 | 1.7 | | -0.5 | 0.36 |
| 0.1–10% | 15 | 0.9 | 1.9 | | -1.0 | 0.079 |
| 10.1–50% | 26 | 1.6 | 1.7 | | -0.1 | 0.44 |
| 50.1–90% | 16 | 2.3 | 3.5 | | -1.2 | 0.49 |
| 90.1–100% | 15 | 6.3 | 2.9 | | 3.4 | 0.027 |

| 2012–2013 | | | | | | |
| --- | --- | --- | --- | --- | --- | --- |
| Stem oiling category | *n* | Mean erosion | Expected when no difference | Change from expectation | | *P*-value |
| 0% | 29 | 1.0 | 0.9 | | 0.1 | 0.089 |
| 0–10% | 16 | 1.4 | 1.0 | | 0.4 | 0.32 |
| 11–50% | 26 | 1.1 | 1.6 | | -0.4 | 0.36 |
| 51–90% | 16 | 2.3 | 1.4 | | 0.9 | 0.13 |
| 91–100% | 16 | 0.8 | 1.5 | | -0.7 | 0.35 |

**Table S3A** Summary data for Figure S2A: total mean erosion (2010–2013) in Louisiana sites only.

| Stem oiling | *n* | Erosion rates (m/yr) | | | *P*-value |
| --- | --- | --- | --- | --- | --- |
| category |  | Mean | SE | Median |  |
| 0% | 15 | 1.4 | 0.5 | 0.8 | 0.10 |
| 0.1–10% | 13 | 1.3 | 0.6 | 0.7 | 0.016 |
| 10.1–50% | 18 | 1.6 | 0.5 | 0.9 | 0.44 |
| 50.1–90% | 16 | 2.1 | 0.6 | 1.5 | 0.12 |
| 90.1–100% | 15 | 4.0 | 1.4 | 1.6 | 0.011 |
| All sites | 77 | 2.1 |  |  |  |

**Table S3B** Summary data for Figure S2B: total, wave adjusted erosion rates for Louisiana sites only.

|  |  | Erosion rates (m/yr) | | |  |
| --- | --- | --- | --- | --- | --- |
| Stem oiling | *n* | Mean erosion rate | Expected mean, no effect of oil | Change from random expectation | *P*-value |
| category |  |  |  |  |  |
| 0% | 15 | 1.4 | 1.4 | 0.1 | 0.14 |
| 0.1–10% | 13 | 1.3 | 2.0 | -0.7 | 0.06 |
| 10.1–50% | 18 | 1.6 | 1.8 | -0.1 | 0.28 |
| 50.1–90% | 16 | 2.1 | 2.9 | -0.8 | 0.063 |
| 90.1–100% | 15 | 4.0 | 2.4 | 1.6 | 0.13 |

**Table S4** Analog of Table S2 with year-specific results for Louisiana only: mean erosion

| 2010–2011 | | | | | | |
| --- | --- | --- | --- | --- | --- | --- |
| Stem oiling category | *n* | Mean erosion | Expected when no difference | change from expectation | | *P*-value |
| 0% | 16 | 1.5 | 1.3 | | 0.2 | 0.34 |
| 0.1–10% | 13 | 1.1 | 1.8 | | -0.7 | 0.081 |
| 10.1–50% | 18 | 1.1 | 1.5 | | -0.4 | 0.40 |
| 50.1–90% | 16 | 1.5 | 2.2 | | -0.7 | 0.11 |
| 90.1–100% | 15 | 3.4 | 1.8 | | 1.6 | 0.040 |

| 2011–2012 | | | | | | |
| --- | --- | --- | --- | --- | --- | --- |
| Stem oiling  category | *n* | Mean erosion | Expected when no difference | change from expectation | | *P*-value |
| 0% | 15 | 1.6 | 1.8 | | -0.2 | 0.32 |
| 0.1–10% | 13 | 1.1 | 2.6 | | -1.5 | 0.15 |
| 10.1–50% | 18 | 2.7 | 2.4 | | 0.2 | 0.41 |
| 50.1–90% | 16 | 2.3 | 4.0 | | -1.6 | 0.21 |
| 90.1–100% | 15 | 6.4 | 3.4 | | 3.0 | 0.068 |

| 2012–2013 | | | | | | |
| --- | --- | --- | --- | --- | --- | --- |
| Stem oiling category | *n* | Mean erosion | Expected when no difference | Change from expectation | | *P*-value |
| 0% | 15 | 1.6 | 1.2 | | 0.4 | 0.21 |
| 0.1–10% | 13 | 1.7 | 1.7 | | 0.0 | 0.52 |
| 10.1–50% | 18 | 1.1 | 1.5 | | -0.4 | 0.48 |
| 50.1–90% | 16 | 2.4 | 2.6 | | -0.2 | 0.40 |
| 90.1–100% | 15 | 2.2 | 2.0 | | 0.2 | 0.36 |

**Figure S1**. A) Box plots of *unadjusted* erosion rates (m/yr) in each stem oiling category for LA sites only. B) Mean excess erosion (m/yr) for each stem oiling category for LA sites only. Excess erosion is the difference between the observed mean erosion for that stem oiling category and the expected mean *wave-adjusted* erosion rate if there were no differences in erosion among the stem oiling categories. The vertical lines are the central 95% randomization distributions for excess erosion in each stem oiling category. When the vertical line does not cross 0, the *p*-value for the comparison of that stem oiling category to the overall erosion rate is less than 0.05.

**Figure S2**. Mean excess erosion (m/yr) by stem oiling category in 2011–2012 and 2012–2013 for LA sites only.
